# Designing policy framework for sustainable development in Next-5 largest economies amidst energy consumption and key macroeconomic indicators

**DOI:** 10.1007/s11356-021-16820-z

**Published:** 2021-10-15

**Authors:** Festus Victor Bekun, Festus Fatai Adedoyin, Daniel Balsalobre- Lorente, Oana M. Driha

**Affiliations:** 1 Department of International Logistics and Transportation, Faculty of Economics and Administrative Sciences, Istanbul Gelisim University, Istanbul, Turkey; 2 Department of Computing and Informatics, Bournemouth University, Poole, UK; 3 Department of Political Economy and Public Finance, Economics and Business Statistics and Economic Policy, University of Castilla-La Mancha, Ciudad Real, Spain; 4 Department of Applied Economics, University of Alicante, Alicante, Spain; 5 Department of Applied Economics, International Economy Institute, Institute of Tourism Research, University of Alicante, Alicante, Spain

**Keywords:** Tourism-economic growth nexus, Foreign direct investment, Energy consumption, Sustainable economic growth: 5 largest economies

## Abstract

Global travel and tourism have enjoyed a significant boost due to the progress in air transport. However, the debate on air transport and the influx of foreign investments and global energy demand on economic development remains questionable. Therefore, this study is an attempt to contribute to the body of knowledge in the energy-tourism-led growth hypothesis literature. For this purpose, a novel approach to the effects of international tourism on economic growth is introduced for the Next-5 largest economies, namely (China, India, Indonesia, Turkey and the USA) between 1990 and 2018. Empirical results reveal a positive connection between foreign direct investment and income levels, electricity production and income levels, as well as between urbanization and economic growth. Moreover, the validation of the environmental Kuznets curve and the halo effect of foreign direct investment on the environmental degradation process provides a shred of more substantial evidence and fitting environmental instruments for policymakers. The empirical results encourage sustainable economic growth in these countries, mainly through the attraction of clean and high-technology foreign investment, the increase of the share of renewable energy sources in the energy mix and the regulation in the tourism industry. The novel contribution of this study to the empirical literature is the unification in the same research of the TLGH and the EKC for the Next-5 largest economies, establishing recommendations for tourism, energy efficiency and environmental correction process.

## Introduction

The World Travel and Tourism Council (WTTC 2018) reported that the tourism industry is a leading sector that drives approximately 3% and subsequently translates into a 10% increase to spur economic expansion. It is, thus, a fundamental driving force of economic growth (Balsalobre-Lorente et al. 2020a, b; Tecel et al. 2020). In this line, several channels are considered, including job creation, foreign investment, new business opportunities, transfer of technology, infrastructure development, energy systems expansion, transportation, human capital, research and development (Schubert et al. 2011; Fahimi et al. 2018). Ever since the United Nations Conference on Trade and Development (UNCTAD 2007) advocated the tourism-foreign direct investment (FDI) interactions to develop the tourism-FDI linkage, it has received increasing attention in the related literature.

**Fig. 1:**
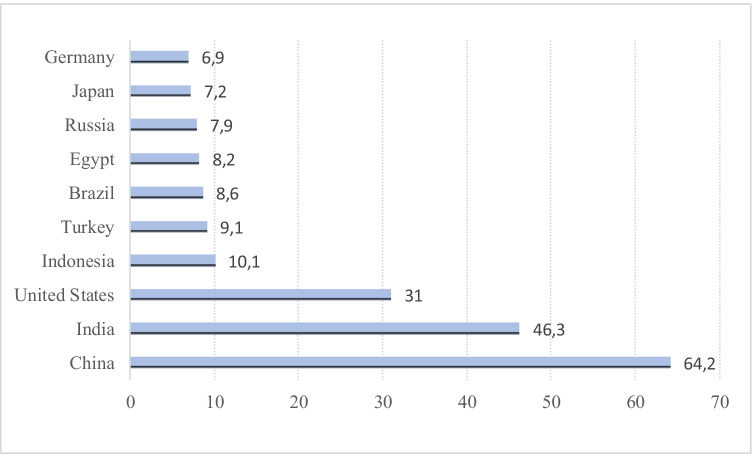
Projections of world’s ten leading economies in 2030 (GDP PPP international dollars, trillion) ( source: IMF (2017); World Economic Outlook, April 2017)

The tourism and service industry literature has drawn particular attention to the phenomenon where tourism drives economic development (Brida et al. 2016; Balsalobre-Lorente et al. 2020a,b), fondly known as the tourism-led growth hypothesis (TLGH). The TLGH offers evidence of economic growth resulting from productive factor inputs and tourism channelled activities (Katircioglu 2009). This paper aims to advance the discussion on the TLGH to highlight the role of the air transport sector as a growth driving force in China, India, Indonesia, Turkey and the USA, considered the Next-5 largest economies (IMF, 2017). These economies are expected to be the largest ones, with over 70% of the global energy usage, reflecting their increased contribution to environmental degradation (IEA 2019) (Fig. 1).

Seeking to explore the empirical validation of TLGH, the linkage between economic growth and the tourism industry is examined while accounting for other macroeconomic indicators. Therefore, a new battery of explanatory variables is proposed to explore the linkage between international tourism (air transport of passengers, hereinafter AT) and economic growth (gross domestic product per capita, hereinafter GDP). Among the selected variables, per capita electricity output (Gw/h per capita, ELEC), foreign direct investment net inflows (FDI), and finally, the share of urban population on GDP between 1990 and 2018. All these new explanatory variables are meant to facilitate the design of more suitable policy recommendations in the context of the Next-5 largest economies. These highlighted macroeconomic variables (FDI, ELEC, AT, URB) have been neglected in the extant literature. This present study is distinct from the previous by holistically modelling the outlined variables, considering the empirical evidence of the TLGH and the EKC in the same survey. This process resonates with the need for sustainable economic growth drivers (SDG-8). Additionally, for the robustness of study coefficients and inferences, a battery of first and second-generational techniques is employed to analyse soundness.

The validation of the TLGH for Next-5 largest economies implies the necessity of articulating suitable policies to promote the advanced tourism industry, promoting clean energy technologies and high tech supplementary services. This study reinforces this evidence, including the validation of the EKC hypothesis (see Appendix). This joint evidence will establish a wide range of recommendations in sustainable development, energy efficiency and environmental correction process, which has not been considered jointly in the previous empirical literature.

So, our study key findings illustrate that air transportation, energy consumption (electricity), increased urban population and influx of FDI are statistically key drivers for sustainable economic growth in the Next-5 economies, validating the TLGH and energy-led growth nexus. Thus, policymakers in these economies will focus on these growth induced sectors and free productive resources to those sectors for increased and sustainable development.

The paper is structured as follows: second section presents the theoretical framework, while the data and methodological process are exposed in third section. Fourth section discusses the empirical results, while fifth section concludes the review with some policy suggestions accordingly.

## Theoretical framework

The relevance and the determinants of economic growth and development have been studied broadly in advanced and emerging economies, being tourism one of its determinants as supported by the TLGH. The importance of tourism has grown exponentially given the multiple benefits tourism present due to employment opportunities and job creation, foreign exchange production, household income and government revenue through multiplier effects, the balance of payments improvements and growth in the number of tourism-promoted government policies (Brida et al. 2016a; Rasool et al. 2021).

Although even before 2002, there were clear hints of interest in the relationship between tourism and economic growth, it was mainly after the first formal reference to the tourism-led growth hypothesis (Balaguer and Cantavella-Jordá 2002) when the empirical evidence started to increase considerably. Based on the previous empirical evidence (Adedoyin and Bekun 2020; Brida et al. 2016a; Balsalobre-Lorente et al. 2021a, b; Pérez-Montiel et al. 2021; Usman et al. 2020; Rasool et al. 2021), there are mainly four hypotheses when exploring the link between tourism and economic growth (Chatziantoniou et al. 2013): (a) two hypotheses based on the unidirectional causality between tourism and economic growth, either from tourism to economic growth (tourism-led economic growth hypothesis, TLGH) or from economic growth to tourism (economic-driven tourism growth hypothesis, EDTH); (b) one hypothesis supporting the existence of bi-directional causality (bi-directional causality hypothesis); and (c) another hypothesis defending no relationship at all (no causality hypothesis). Still, the results are mixed and sample-dependent (Kumar Mitra 2018) and even conflicting despite the homogeneity of the research methods chosen (Katircioğlu 2014; Roudi et al. 2019; Rasool et al. 2021). While, TLGH defends that tourism, through a series of benefits, can promote economic growth via different routes (Schubert et al. 2011; Brida et al. 2016a; Shahzad et al. 2017; Balsalobre-Lorente et al. 2020a, b; Tecel et al. 2020), mastering the specialized literature, little evidence was found in line with EDTH (Narayan 2004; Oh 2005; Payne and Mervar 2010). The stability of well-designed economic policies, governance structures and physical and human capital investments seem to promote tourism growth (Payne and Mervar 2010). A series of development activities would be created with such context, which can considerably contribute to tourism development (Rasool et al. 2021). On bi-directional causality grounds, some evidence was found (Lee and Chang 2008; Ridderstaat et al. 2014). For this kind of reciprocal tourism-economic growth relationship, policymakers are expected to simultaneously design agendas to promote both areas. Moreover, some studies found no support to any of these hypotheses, defending the insignificant relationship between tourism and economic growth (Katircioglu 2009; Po and Huang 2008; Tang 2013).

Adedoyin and Bekun (2020) explored the nexus between tourism, energy consumption, CO_2_ emissions and urbanization for seven tourism-dependent countries from 1995 to 2014 using a panel VAR approach complemented with panel causality analysis. The study supports a feedback causality between tourism and urbanization. The study also employed impulse response analysis to show the elasticity shock of each variable on another, and the impulse response function shows that tourism responds positively to shocks in urbanization throughout the study time horizon. In conclusion, this study draws pertinent energy and tourism policy implications for sustainable tourism on the panel over their growth path without compromising for the green environment.

Lo et al. (2018) confirmed a direct connection between air transport and economic growth in Italy during 1997–2011, and Morazzo et al. (2010) found the same result in Brazil between 1966 and 2006. Considering the bond between energy use and economic growth, the rise in energy consumption is expected to imply increases in economic growth and invariably more levels of carbon emissions (for more details, see Appendix).

Kalayci and Yanginlar (2016) found that air transportation impacts economic growth more than FDI. Air transport contributes to regional economic development and stability (Fleming and Ghobrial 1994; Mody and Wang 1997; Helling 1997; Banister and Berechman 2003; Hong et al. 2011; Mukkala and Tervo 2013 Kalayci1 and Yanginlar 2016). Helling (1997) handled the employment dimension of the economy and proved how transportation systems also ensure job accessibility and quality of life. Banister and Berechman (2003) draw a general perspective of the linkage between economic development and air transport logistics, showing that transportation infrastructure enhancements reduce harmful effects, increasing traffic volume. In the same line, Hong et al. 2011) concluded that advanced air infrastructures provide more FDI, generating a significant economic development catalyst. Çiftçioğlu and Sokhanvar (2021) investigated the relationship between tourism specialization, domestic investment rate and sustainable economic development. They confirm that further specialization in tourism was likely to promote economic growth in Macao and Malaysia. Rasool et al. (2021) conclude that BRICS should design tourism policies able to shove economic growth.

Moreover, empirical studies recognize two main ways to connect FDI and economic growth (Banister and Berechman 2003; Adams 2009; Li et al. 2017; Rao et al. 2020). First, promoting local capital and enhancing efficiency through the transfer of new technologies can reduce the gap between economic growth and FDI. Secondly, FDI presents both benefits and costs, which impact is determined by the country-specific conditions in general and the policy environment (e.g. ability to diversify, absorption capacity, targeting of FDI and opportunities). So, the initial country’s conditions alter the connection between FDI and economic growth (Trevino and Upadhyava 2003; Ajayi 2006; Alfaro et al. 2004b, a). FDI is more likely to promote economic development in open economies (Trevino and Upadhyaya 2003). Alfaro et al. 2004b, a) argued that the growth-enhancing effect of FDI requires a developed financial system. Rao et al. (2020) found that FDI positively influences growth in South-East Asia (SEA) and South Asia (SA) during 1980–2016. Furthermore, Rao et al. (2020) alluded to the fact that improving governmental financial assistance to the private sector for domestic investment is essential to spur FDI flows positively. This process will attract and absorb the benefits of complementing FDI flows and sustaining higher economic growth in the long run.

By contrast, some studies suggest that the negative impact of FDI on economic growth is the consequence of a powerful monopolistic industrial infrastructure that prevents local industry from growing (Bornschier and Chase-Dunn 1985; Rhagavan’s 2000; Ajayi 2006). Hence, it is necessary to consider the potentially pernicious effects FDI might have over host countries due to the climbed monopolization of local industries, totally reliant on FDI, generating unemployment and inequality. So, it is expected that local investment would be stimulated by FDI, with positive externalities resulting from technology transfer and spillovers being related to the tourism industry (Carkovic and Levine 2002; Ajayi 2006; Banister and Berechman 2003).

Other studies assume a positive impact of FDI on economic growth (Ndikumana and Verick 2008; Lumbila 2005), but others suggest a nonsignificant or a negative effect of FDI on economic growth (Akinlo 2004; Ayanwale 2007; De Mello 1999). De Mello (1999) found that FDI exerted a harmful impact on economic growth for a selected panel of non-OECD countries due to reduced total factor productivity in host countries due to foreign business. In the same line, Alfaro (2003) explored the impact of FDI on economic growth in the primary, manufacturing and services sectors, showing that FDI benefits vary significantly across industries. While FDI in the primary sector tended to impact growth negatively, it appears favourable for the manufacturing industry and ambiguous in the service sector. Identical results were exhibited by Habiyaremye and Ziesemer (2006). They concluded that the overall level of capital investment does not affect economic growth when most of the capital is in the primary sector for Sub-Saharan African countries. Akinlo (2004) found that the impact of FDI on the Nigerian economy was not significant.

The ascending levels of FDI are not always accompanied by a similar level of economic growth in host countries, as a consequence of the presence of pernicious effects of foreign enterprises in local business (Bannò and Redondi 2014; Tang et al. 2007; Katircioglu 2011; Samimi et al. 2017; Tomohara 2017). Tomohara (2017) showed a direct connection between international tourism and FDI. Promoting active tourism-related policies creates new business opportunities; expands the tourism infrastructures; and develops accommodation, restaurants or transportation infrastructures (Tang et al. 2007; Katircioglu 2011; Samimi et al. 2017). Regarding the relationship between air transport and the FDI nexus, some studies confirm that it generates positive externalities over the other economic sectors (Tiwari 2011). Tolcha et al. (2020) validated this process, suggesting that air transport enhances economic growth under the TLGH scenario. Sinclair (1998) illustrated multiplier effects in the tourism industry, integrating economic systems and their development strategies generating foreign exchange earnings or technical advances. Kasarda and Green (2005) concluded that air transport, as a dynamizing industry of economic development, tends to lead to globalization processes and the consequences of FDI, while the tourism industry can relieve foreign exchange constraints, reducing the pernicious effects of FDI on economic growth (Nowak et al. 2007). International tourism and FDI inflows have generated detectable beneficial impacts on the economy of Estonia in the last decades. However, recently, poor global market conditions caused mainly by the trade war and COVID-19 pandemic have been a potential threat to these two factors.

In line with this evidence, air transport, as a proxy of international tourism, is expected to catalyse the technological transference and innovations processes of FDI, boosting the reception of goods and services leading in turn to economic growth, in line with previous empirical evidence (Borensztein et al. 1998; Alfaro et al. 2004b, a; Iamsiraroj 2016; Newman et al. 2015).

Furthermore, in empirical tourism literature, electricity output and the share of the urban population have been employed as control variables to avoid omitted variable biases in econometric modelling. The analyses of these variables as channels of economic growth complement the study offering advanced research with relevant implications for selected Next-5 countries. Substantial evidence of direct stimulation of economic growth through energy consumption is available (Tang and Abosedra 2012, 2014; Tang 2008; Tang and Tan 2013a; b; Tang 2013; Apergis and Tang 2013; Liang and Yang 2019; Adebayo 2021). Liang and Yang (2019) concluded that urbanization promotes economic growth by accumulating physical capital, knowledge capital and human capital, in the case of China. Adebayo (2021) showed that urbanization, globalization and energy usage trigger economic growth in Japan from 1970 to 2015.

Thus, this study differs from the extant literature in the following ways: (1) The central proposal of this study is to validate the TLGH in the Next-5 largest countries, which have not been documented in the extant literature. Additionally, to our knowledge, it has not been considered holistically in the literature. (2) The additional contribution beyond scope but the border around the careful choice of the model covariates, which aligns with the United Nations Sustainable development goals (UNSDGs) of access to energy, responsible consumption (UN-SDG-7,13), economic growth (SDG-8) and climate change mitigation (SDG-13) in the context of the selected panel while accounting for key covariates. This contribution is supported by analysing the linkage between economic growth and carbon emissions to validate the environmental Kuznets curve (EKC) for the selected Next-5 panel (see Appendix Table 1 for results). Finally, it is worth mentioning that this study goes beyond a non-linear linkage between tourism and the pollution halo hypothesis or the energy-led growth hypothesis by offering a compact empirical contribution through a triple-lens.

## Empirical methodology and econometric results

This study aims to validate the tourism-led growth hypothesis (TLGH) concept, where the environmental Kuznets curve (EKC) is used as a complementary analysis for the Next-5 largest economies between 1990 and 2018. Coincidentally, the investigated blocs are highly tourism destinations and thus necessitate the need to explore the role of international tourism arrival on their respective economic growth. However, the need for environmental sustainability is also investigated in an EKC framework. Hence, the empirical results will provide evidence of the positive effect of air transport as a proxy of international tourism on economic growth. This study also considers energy production, FDI and urbanization process an essential control variable for policy construction after the previous studies (Dogru and Bulut 2018; Hu et al. 2019; Balsalobre-Lorente et al. 2021a).

Additionally, beyond the empirical support as highlighted, the empirical motivation for this study stem forms the tourism-induced growth hypothesis (TLGH), where tourism is considered a catalyst for economic growth. In our study case, tourism is estimated by air transportation after the study of Balsalobre-Lorente et al. 2021a). However, tourism is measured by international tourism arrivals and receipts in the tourism-energy literature, which have received criticism in the extant literature.

Furthermore, the theoretical underpinning of the present study draws strength to form the carbon-income function backup by the EKC concept (see Appendix). That outlines the tradeoff between economic growth (tourism development) and the quality of the environment. A holistic study of this phenomenon with key macroeconomic variables like FDI inflows and energy consumption is pertinent for policy construction for the Next-5 largest economies, which is the study’s motivation, which has not been well documented in the tourism-energy and growth literature. To check our main hypotheses, we propose Eq. 1:

1 $${LGDP}_{it}={\beta }_{0}+{\beta }_{1}L{AT}_{it}+{\beta }_{2}{LELEC}_{it}+ {\beta }_{3}{LFDI}_{it}+ {\beta }_{4}{LURB}_{it}{+ \varepsilon }_{it}$$

This study employs the annual information (Table 1) facilitated by the World Bank database and the International Energy Agency (WDI 2021; IEA 2021).

**Table 1:** Main statistics and Correlation Matrix

	LCO2	LGDP	LAT	LELEC	LFDI	LURB
Mean	1.127421	8.128094	17.99744	-6.352899	22.07853	3.886120
Median	0.900568	8.024190	17.89954	-6.718830	23.74419	3.923853
Maximum	3.010128	11.05083	20.60563	-4.237372	26.96048	4.409836
Minimum	-0.499226	5.707638	14.96608	-8.622168	-22.23847	3.240520
Std. Dev	1.063394	1.584228	1.567543	1.264415	8.411070	0.390221
Skewness	0.508310	0.329691	0.217040	0.437006	-4.651159	-0.165748
Kurtosis	2.114456	1.958833	1.787533	2.028628	24.12271	1.572901
Jarque–Bera	10.98197	9.176169	10.02011	10.31590	3218.408	12.96845
Probability	0.004124	0.010172	0.006671	0.005753	0.000000	0.001527
Sum	163.4760	1178.574	2609.629	-921.1704	3201.387	563.4874
Sum Sq. Dev	162.8362	361.4081	353.8354	230.2194	10,187.44	21.92721
Observations	145	145	145	145	145	145
Correlation Matrix						
	LCO2	LGDP	LAT	LELEC	LFDI	LURB
LCO2	1.000000					
LGDP	0.921742	1.000000				
LAT	0.829429	0.766478	1.000000			
LELEC	0.978758	0.943689	0.827413	1.000000		
LFDI	0.326973	0.327280	0.424077	0.364779	1.000000	
LURB	0.835838	0.934040	0.541894	0.844345	0.169336	1.000000

Sources: World Bank (2021), IEA (2021)

The sample includes the period 1990–2018, and it is based on the fact that these countries are considered the Next-5 largest economies (World Economic Forum 2019; Farhani and Balsalobre-Lorente 2020). $${LGDP}_{it}$$ stands for the logarithm of GDP per capita (current US$), $$L{AT}_{it}$$ is the natural logarithm of air transport, passengers carried, as a proxy of tourism, in line with Balsalobre-Lorente et al. (2021a). The consideration of air transport is mainly employed cause of the increased availability of data (WDI, 2021), which allows us to explore all more extensive range of years and, consequently, a more robust analysis. The linkage between $${LFDI}_{it}$$ and $${LGDP}_{it}$$ defines the nature of host business and foreign business's impact on the local economy (Trevino and Upadhyava 2003; Ajayi 2006; Alfaro et al. 2004b, a). $${LELEC}_{it}$$ is the logarithm of per capita Gw/h electricity output (Lee and Brahmasrene 2013; Salahuddin et al. 2018). This variable traditionally is included, in the empirical literature, as a fundamental driving force of economic growth, contributing to avoiding omitted variables. Finally, this study also explores the role of urbanization ($${LURB}_{it}$$) on economic growth (Liu and Bae 2018) in selected panel.

Our study checks Chudik and Pesaran (2013) cross-sectional dependence (Table 2).

**Table 2:** Cross-sectional dependence and second-generation panel unit root tests

Variables	CD statistics	CIPS		CADF	
		Level	1st Difference	Level	1st Difference
LCO2	10.727*	− 1.857	− 4.388*	− 2.084	− 4.388*
LGDP	16.990*	− 0.519	− 4.910*	− 1.981	− 3.511*
LAT	17.020*	− 1.478	− 4.842*	− 2.215	− 3.367*
LELEC	17.003*	− 1.604	− 4.306*	− 1.500	− 4.306*
LFDI	10.004*	− 1.825	− 4.672*	− 2.031	− 3.741*
LURB	17.019*	− 1.023	− 2.178***	− 2.239	− 2.265***

*, ** and *** indicate statistical significance at 1%, 5% and 10% respectively

Table 2 shows Chudik and Pesaran (2013) test results, confirming the existence of cross-sectional dependence. Table 2 also evaluates the stationarity properties of selected variables through CIPS and CADF second-generation unit root tests. The CIPS and CADF tests evaluate the selected variables’ stochastic properties and avoid cross-sectional problems, indicating if the chosen variables are cointegrated in order 1.

Confirmed that selected variables are stationary at the first difference, I(1) (see Table 2), in the next step, we check the long-run relationship of the panel, through the selected Kao (1999), Johansen-Ficher (1991) and Westerlud’s (2007) panel cointegration tests. Kao’s (1999) test uses cross-section intercepts and homogeneous coefficients on the first stage regressors. Fisher-Johansen’s (1991) cointegration test combines individual tests and connecting tests from individual cross-sections.

The Westerlund (2007) cointegration test is applied for checking the cointegration properties in the presence of cross-sectional dependence.

Table 3 results confirm a long-run relationship among selected variables, revealing a significant cointegrating association between 1990 and 2018. Once we have determined the existence of a long-run relationship between the variables, we apply the FMOLS and DOLS estimation methodology (Phillips and Hansen 1990). These econometric techniques offer serial correlation and endogeneity adjustment due to cointegrating relationships (Balsalobre-Lorente et al. 2021a, b). We previously explored the causal connection among selected variables applying the Dumitrescu and Hurlin (2012) panel causality test (Table 4). Dumitrescu and Hurlin 2012) causality test offers reliable results for small and unbalanced samples and with cross-sectional dependence.

**Table 3:** Kao and Johansen Fisher panel cointegration tests

a)Kao cointegration test					
	*t*-Statistic	Prob			
ADF	− 2.187859*	(0.0143)			
Residual variance	0.001043				
HAC variance	0.001102				
b)Johansen Fisher panel cointegration test Unrestricted cointegration rank test (trace and maximum eigenvalue)					
Hypothesized no. of CE (s)	Fisher stat. # (from trace test)		Fisher stat. # (from max-Eigen test)		
	*t*-Statistic	Prob		*t*-Statistic	Prob
*r* $\le 0$	166.3*	(0.0000)		85.79*	(0.0000)
*r* $\le$ 1	97.04*	(0.0000)		42.38*	(0.0000)
*r* $\le$ 2	63.50*	(0.0000)		29.10*	(0.0012)
*r* $\le$ 3	42.06*	(0.0000)		25.23*	(0.0049)
*r* ≤ 4	26.18**	(0.0035)		23.83*	(0.0081)
c)Westerlund (2007) cointegration test					
Test	Value	*Z* value		*p* Value	Robust *p* value
Gt	− 3.090*	− 0.184		(0.427)	(0.000)
Ga	− 2.323	4.111		(1.000)	(1.000)
Pt	− 5.933*	0.297		(0.617)	(0.000)
Pa	− 2.303	3.316		(1.000)	(1.000)

*, ** and *** indicate statistical significance at 1%, 5% and 10% respectively; # Probabilities are computed using asymptotic Chi-square distribution

Table 4 presents Dumitrescu and Hurlin’s (2012) test results. The DH test reflects a bi-directional causality between *LAT*_*it*_ to *LGDP*_*it*_ (Baker et al. 2015; Kalayci1 and Yanginlar 2016; Saidi and Hammami 2017; Rasool et al. 2021). This result suggests feedback between air transport and economic growth, establishing that economic growth and air transport have reciprocal effects. Consequently, the air transportation industry will ensure more extensive socioeconomic interests via its potential to permit exact types of local economy activities (Kalayci1 and Yanginlar 2016; Rasool et al. 2021). Table 4 also shows a unidirectional causality running from *LGDP*_*it*_ to *LFDI*_*it*_ (Abdouli and Omri 2020). This evidence suggests that GDP is a good predictor for GDP growth in the Next-5 largest economies. Such policymakers in those economies will create an enabling environment for attracting FDI inflow driven by a robust economy. Table 5 presents the fully modified ordinary least square (FMOLS) (Phillips and Hansen 1990) and dynamic ordinary least square (DOLS) (Saikkonen 1991; Stock and Watson 1993) econometric techniques. These techniques are appropriate for serial correlation and endogeneity problems. The DOLS methodology is built to be an asymptotically efficient estimator and eliminate feedback in the cointegrating system. Additionally, as outlined by the study of Narayan and Narayan 2010), the DOLS technique is built on the orthogonality in the cointegrating equation error term, offering an asymptotically efficient estimator that eliminates feedback in the cointegrating system. The DOLS method is also suitable for samples with lesser size by disregarding inaccuracy caused by sample bias.

**Table 4:** Pairwise Dumitrescu-Hurlin panel causality tests

Null hypothesis		Causality	W-Stat	Zbar-Stat	Prob
LGDP does not homogeneously cause LCO2		LGDPAT → LCO2	6.53326	3.98384	(7.E-05)
LCO2 does not homogeneously cause LGDP			3.02513	0.75859	(0.4481)
LAT does not homogeneously cause LCO2		LAT → LCO2	5.32975	2.87738	(0.0040)
LCO2 does not homogeneously cause LAT			2.68578	0.44661	(0.6552)
LELEC does not homogeneously cause LCO2		LELEC → LCO2	6.10484	3.58997	(0.0003)
LCO2 does not homogeneously cause LELEC			3.85031	1.51723	(0.1292)
LFDI does not homogeneously cause LCO2		LFDI → LCO2	2.18230	-0.01627	(0.9870)
LCO2 does not homogeneously cause LFDI			7.90446	5.24448	(2.E-07)
LURB does not homogeneously cause LCO2		LURB ↔ LCO2FDI	11.2560	8.32575	(0.0000)
LCO2 does not homogeneously cause LURB			5.48523	3.02032	(0.0025)
LAT does not homogeneously cause LGDP		LGDP ↔ LAT	4.39134	4.49627	(7.E-06)
LGDP does not homogeneously cause LAT			3.28444	2.99012	(0.0028)
LELEC does not homogeneously cause LGDP		LELEC → LGDP	6.05574	3.54483	(0.0004)
LGDP does not homogeneously cause LELEC			2.97589	0.71333	(0.4756)
LFDI does not homogeneously cause LGDP		LGDP → LFDI	2.09283	-0.09853	(0.9215)
LGDP does not homogeneously cause LFDI			8.33962	5.64454	(2.E-08)
LURB does not homogeneously cause LGDP		LURB → LGDP	6.21205	3.68854	(0.0002)
LGDP does not homogeneously cause LURB			3.74613	1.42146	(0.1552)
LELEC does not homogeneously cause LAT		LELEC → LAT	4.46359	2.08106	(0.0374)
LAT does not homogeneously cause LELEC			2.08922	-0.10184	(0.9189)
LFDI does not homogeneously cause LAT		LAT ↔ LFDI	3.19733	0.91691	(0.3592)
LAT does not homogeneously cause LFDI			9.23878	6.47119	(1.E-10)
LURB does not homogeneously cause LAT		LAT ↔ LURB	11.1174	8.19830	(2.E-16)
LAT does not homogeneously cause LURB			13.1535	10.0703	(0.0000)
LFDI does not homogeneously cause LELEC		LELEC → LFDI	1.76926	-0.39600	(0.6921)
LELEC does not homogeneously cause LFDI			6.95274	4.36949	(1.E-05)
LURB does not homogeneously cause LELEC		LELEC ↔ LURB	5.67591	3.19562	(0.0014)
LELEC does not homogeneously cause LURB			9.85199	7.03496	(2.E-12)
LURB does not homogeneously cause LFDI		LFDI ↔ LURB	8.92355	6.18139	(6.E-10)
LFDI does not homogeneously cause LURB			5.40860	2.94987	(0.0032)

*, ** and *** indicate statistical significance at 1%, 5% and 10% respectively. Here, ≠ denotes null hypothesis of “does not Granger cause”. Rejection of the null hypothesis suggests causal interaction between the considered pair of variables

**Table 5:** FMOLS and DOLS econometric results

Dependent variable: $LGDPPC$		
	Equation 1	
	FMOLS	DOLS
LAT	0.146888* [15.68997] (0.0000)	0.121651** [2.015483] (0.0479)
LELEC	0.466072* [59.67585] (0.0000)	0.651301* [22.52070] (0.0000)
LFDI	0.008336* [4.863771] (0.0000)	0.019739* [6.671536] (0.0000)
LURB	2.126180* [56.02697] (0.0000)	2.414388* [8.302045] (0.0000)
*R*-squared	0.970254	0.997954
Adjusted *R*-squared	0.969598	0.996001
SE of regression	0.274215	0.098774
Long-run variance	0.023227	0.005428
Mean dependent var	8.160835	8.163443
S.D. dep var	1.572671	1.562040
Sum squared resid	10.22635	0.643920

*, ** and *** indicate statistical significance at 1%, 5% and 10% respectively. We have checked homogeneous and heterogeneous (sandwich) variance in FMOLS estimation to check the robustness of econometric results. Both estimations processes offer the same results

The econometric results evidence a direct impact (*β*_*1*_ > 0) of *LAT*_*it*_ on *LGDP*_*it,*_ validating the TLGH (Jiao et al. 2019; Mitra 2019; Etokakpan et al. 2019; Balsalobre-Lorente et al. 2021a) for selected Next-5 countries during the period 1990–2018. Besides, the empirical output also confirms a positive (*β*_*2*_> 0) linkage between energy use (*LELC*_*it*_) and economic growth. The connection between FDI and economic growth presents a positive sign (*β*_*3*_ > *0*), revealing that the foreign industry enhances the host economic systems (Li et al. 2017). Finally, the share of the urban population (*LURB*_*it*_) enhances economic growth (*β*_*4*_> 0).

## Discussion of empirical results

**Fig. 2:**
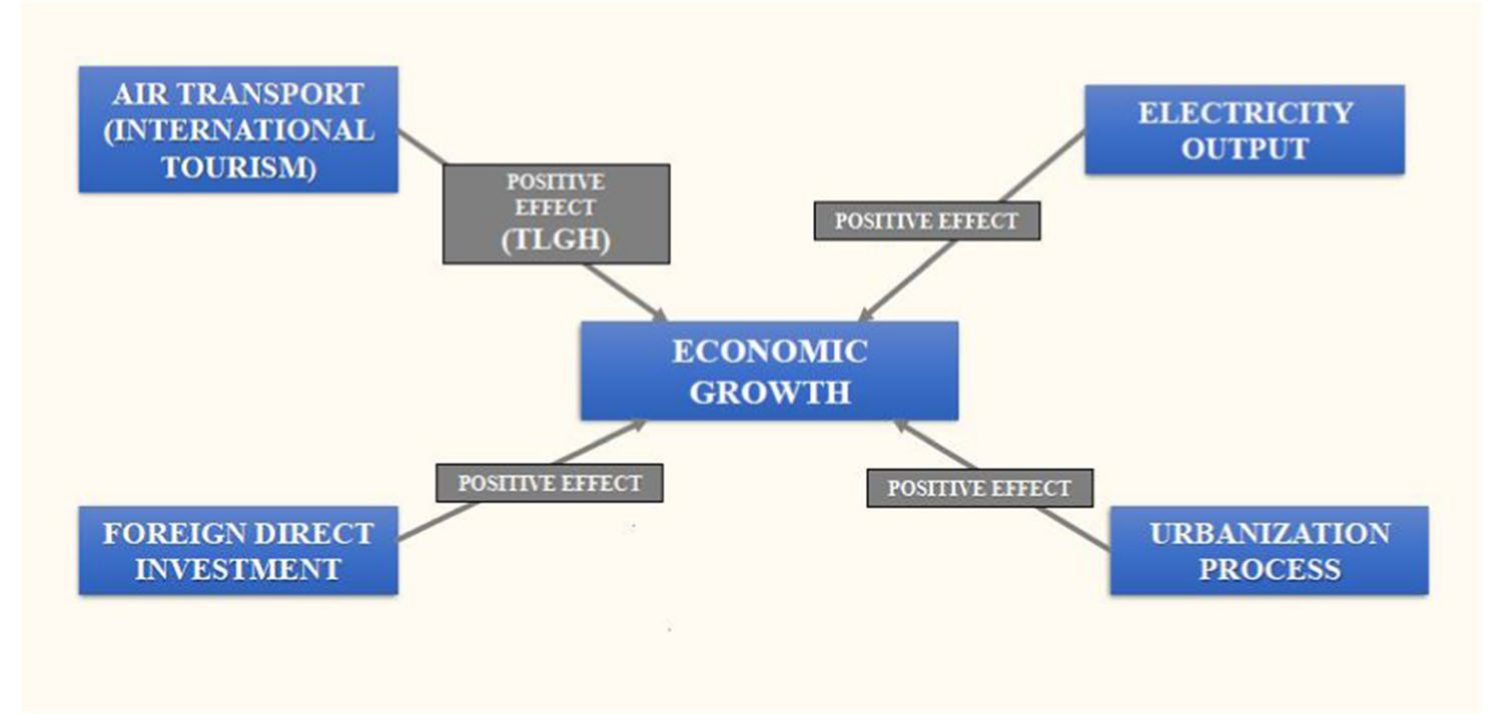
Graphical abstracts of proposed models ( source: prepared by authors)

This study introduces empirical advances in the TLGH empirical literature, considering a new battery of variables that include not only the effect of air transport (as a proxy of international tourism) on economic growth (Fig. 2) for the selected Next-5 largest economies.

The econometric results reveal not only the validation of the TLGH for the selected Next-5 largest countries between 1990 and 2018. These results also confirm a direct impact of electricity output, FDI and urbanization on economic growth. This evidence confirms the energy-induced growth hypothesis. In other words, the economic growth of the Next-5 largest economies is positively induced by energy consumption. The energy-led growth hypothesis is insightful for the Next-5 largest economies as global energy demand increases due to international travel and interconnectedness via the FDI channel.

**Fig. 3:**
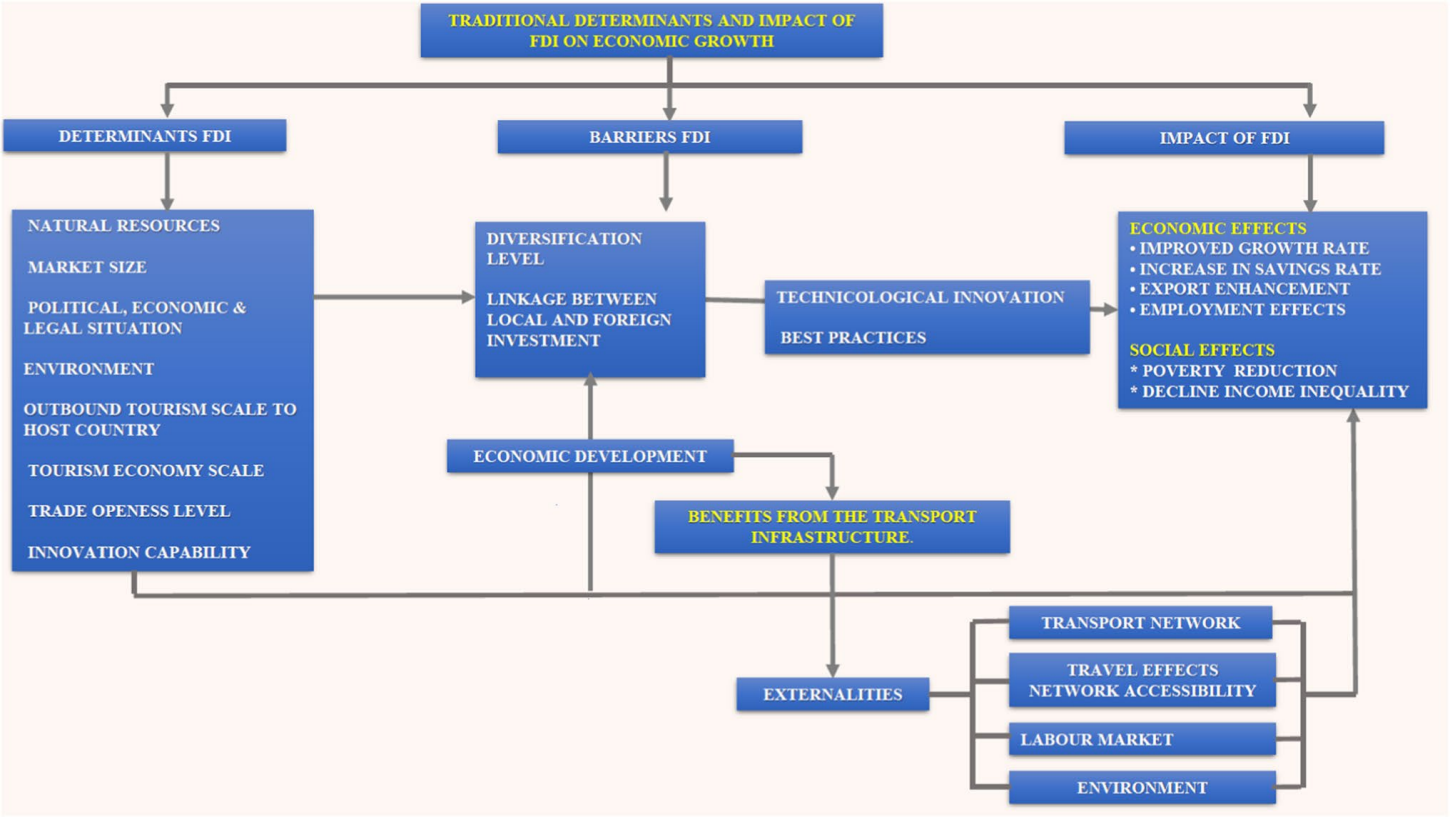
Linkage between economic growth, FDI and tourism (transportation) ( source: prepared by authors based in Banister and Berechman (2001), Adams (2009) and Li et al. (2017))

The direct effect of FDI on income in host countries implies that these countries’ initial conditions and the nature of FDI will significantly boost domestic competitiveness, enhance skills and invariably lead to social and economic gains (Banister and Berechman 2001; Adams 2009; Li et al. 2017). The empirical results support that the diversification level, and the absorptive capacity of local firms, can provide new business opportunities for connexions between host and foreign investors and a targeted FDI approach (Fig. 3).

Some studies (Banister and Berechman 2001; Adams 2009; Li et al. 2017) suggest that FDI contributes to the appearance of positive externalities, like the expanding labour and the expansion of related tourism industries, or the diversification process and the transition from primary to the manufacturing and services sector (UNCTAD 2007; 2008; Brida et al. 2016a; Dogan et al. 2017; Risso 2018; Etokakpan et al. 2019). Our empirical results reveal that these Next-5 largest economies need to invest in improving energy efficiency and regulate necessary environmental protection policies for the tourism industry in specific and promote trading activities and the attraction of clean business. Hence, the selected countries will require to attract diversified and higher value-added FDI. In this regard, these countries should reduce their barriers to FDI effectiveness through investment in tourism-related activities, human capital, more efficient energy processes or local productive capacity. In this sense, these economies’ capability to promote specific regulations for attracting advanced FDI will enhance host capacities to generate positive spillovers and economic growth. This process will also reduce dirty inputs and slow down economic growth in the early stages of development (Balsalobre and Álvarez 2017; Balsalobre-Lorente et al. 2018; Shahbaz et al. 2019a, b). Finally, emphasizing energy consumption due to economic growth, urbanization processes also evidence a positive linkage with economic growth in the selected panel. Else, aimed to provide sustainable development objectives, our study also provides evidence of the EKC for the selected panel as supplementary material (see Appendix).

## Concluding remarks

The last decade has demonstrated the need to examine sustainable development in a broader context, particularly for large and emerging economies. Thus, this study aims to validate or refute the tourism-led growth hypothesis (TLGH) in the Next-5 leading economies between 1990 and 2018. Since we confirm Chudik and Pesaran (2013) cross-sectional dependence, we apply the unit root tests of CIPS and CADF for validating that all variables are cointegrated I(1). Kao (1999), Johansen (1991) and Westerlund’s (2007) panel cointegration tests validate the long-run linkage among selected variables. The Dumitrescu-Hurlin Granger explores the causality direction of the selected variables. This study validates the TLGH applying the FMOLS and DOLS cointegration econometric techniques, which tackle the endogeneity and serial correlation issues. The empirical results confirm the air transport sector’s inevitability advancement to reach a developed and sustainable tourism industry in selected Next-5 countries.

Additionally, electricity output, FDI and urbanization process directly impact economic growth. Following empirical results, these Next-5 leading countries’ governments need to adopt clean energy regulations to attract sustainable tourism and reduce energy dependency as conservative energy policies might hurt economic growth given the reliance of these economies on their energy sector. In other words, these countries need to consolidate the developed tourism industry, increase clean technologies and use renewable sources to avoid environmental degradation processes (these consequences are reinforced in the Appendix section, where we include the validation of the EKC for selected Next-5 largest economies).

The current study examines and contributes to the extant literature on the TLGH, energy-induced and FDI-growth nexus literature while accounting for macroeconomics indicators like urban population size and international travel proxy by air transport Next-5 largest economies. The positive effects of FDI on economic growth emerges from the type of FDI and positive externalities. So, policymakers need to implement measures for attracting high-tech FDI. Our empirical results confirm the advanced development phase in selected countries and how it enhances economic growth.

Otherwise, budgetary effort to promote the attraction of high-tech FDI could provide additional revenues and provide social welfare schemes. These measures are prominent in developing economies where economies thrive on the primary sector, like agriculture and mining. Subsequently, the economic growth trajectory moves to the service stage evidence among developed economies, which have embraced clean technologies, emphasizing environmental consciousness for sustainable growth. However, FDI has immense benefits to the host economy. However, the development process must start from within, through a substantial investment in human capital accumulation and a significant increase in infrastructure provision. A solid basis for a diversified production system can be established, which will serve as a channel to promote technological learning and technology diffusion.

Moreover, the econometric results confirm that the urbanization process enhances economic growth. Consequently, these countries need to develop strategies to promote infrastructures that attract tourism and FDI, coordinating business with local businesses. Thus, our study confirms that the tourism industry could catalyse FDI, creating new employment opportunities, generating tax revenues and gaining foreign exchange earnings. This study also advances, in the empirical literature, testing the dampening effect between FDI and air transport over economic growth in the Next-5 largest economies. The empirical evidence also confirms that air transport and subsidiary tourism-related activities intensify income levels. At this stage, policymakers need to promote suitable measures aimed to enhance the tourism industry. For instance, these countries need to promote renewable energy sources and attract foreign high-tech businesses. The reduction of dirty tourism-related activities will generate new opportunities to improve the Next-5 economies’ local business and tourism industry.

Therefore, it is pertinent for future studies to consider exploring other regions or blocs using disaggregated data for emerging divides like BRICS and SSA as outcomes could differ substantially. Furthermore, future studies should focus on the analysis of trade openness and sustainable growth. The analysis of the globalization process and the impact of COVID-19 over these economies could generate relevant advances in the empirical literature.

## Figures and Tables

**Fig. 4:**
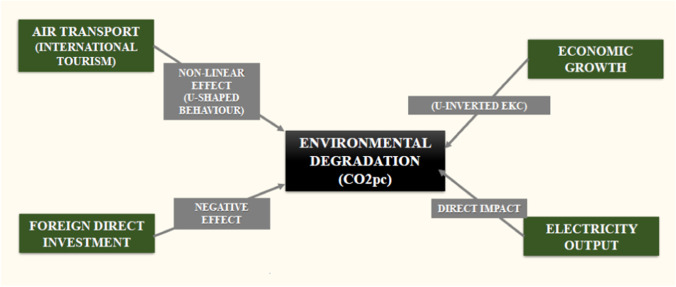
Graphical abstract of validation of the EKC for selected Next-5 largest economies ( source: prepared by authors)

## Data Availability

Available under request.

## Appendix

In this section, we validate the EKC aimed to support sustainable objectives for selected Next-5 largest economies.

In this section, we apply the FMOLS and DOLS econometric technique to validate the EKC hypothesis for selected Next-5 largest economies between 1990 and 2018. This study is in line with El Menyari (2021), who explored the effects of international tourism, electricity consumption and economic growth on CO_2_ emissions in North Africa over the period 1980–2014.

$${LCO2}_{it}={\beta }_{0}+{\beta }_{1}L{GDP}_{it}+{\beta }_{2}{LGDP2}_{it}+ {\beta }_{3}{LAT}_{it}+ {\beta }_{4}{LAT2}_{it}{++ {\beta }_{5}{LELEC}_{it}+ {\beta }_{6}{LFDI}_{it}+ {\beta }_{7}{LURB}_{it}+ \varepsilon }_{it}$$

Table 6 reveals a non-linear influence of economic growth and tourism on per capita carbon emissions in the selected Next-5 economies. The econometric results validate the EKC for the selected sample. Further empirical results give credence to the energy induced emission level in the invested bloc. A similar trend is seen as energy consumption (fossil-fuel base), and FDI inflow increases CO_2_ emission in the Next-5 economies. This complimentary analysis also confirms the detrimental effect of FDI inflow on environmental quality, supporting the pollution haven hypothesis. This outcome resonates with the findings of Joshua et al. (2020) for the case of South Africa, where FDI inflow and urban population spur pollution status in South Africa. However, the higher-income level increases environmental degradation in the next Next-5 largest panel under review given their economic progress. Thus, policymakers are encouraged to pursue clean FDI attraction and sound macroeconomic policies that disentangles urbanization from increased CO_2_ emission. The empirical results show that electricity consumption positively affects CO_2_ emissions, assuming the necessity of reducing the share of fossil sources in the energy mix for selected countries. In contrast, FDI negatively relates to CO_2_ emissions, confirming clean and high-tech FDI inflows in host countries. Urbanization processes also contribute to reducing environmental pressure.

**Table 6:** Air transport-economic growth-CO_2_ linkage: the validation of the EKC for selected Next-5 largest economies (1990–2018)

Dependent variable: LCO2		
Variable	FMOLS	DOLS
LGDP	0.710551* [21.49899] (0.0000)	0.778356* [4.809770] (0.0000)
LGDP^2	− 0.046205* [− 24.62176] (0.0000)	− 0.045210* [− 4.661169] (0.0000)
LAT	− 0.911228* [− 12.65488] (0.0000)	− 1.244359* [− 3.621750] (0.0005)
LAT^2	0.026050* [12.30153] (0.0000)	0.035454* [3.701088] (0.0004)
LELEC	1.102632* [31.31435] (0.0000)	1.139858* [9.919698] (0.0000)
LFDI	− 0.000988* [− 2.845544] (0.0052)	− 0.002732** [− 2.324809] (0.0223)
LURB	− 1.591031* [− 18.02299] (0.0000)	− 2.136095* [− 5.859213] (0.0000)
*R*-squared	0.998368	0.999109
Adjusted *R*-squared	0.998228	0.998668
SE of regression	0.044362	0.038457
Long-run variance	0.000503	0.001470
Mean dependent var	1.141960	1.141960
S.D. dependent var	1.053883	1.053883
Sum squared resid	0.251903	0.137539

Note: *, ** and *** indicate statistical significance at 1%, 5% and 10% respectively.

In short, Fig. 4 provides a comprehensive schematic analysis of the relationship between sustainable economic growth, international tourism and environmental degradation, highlighting the TLGH and EKC analysis.

Based on the survey of extant literature, we claim that this study is the first of its kind to be conducted that comprehensively explored the EKC and TLGH phenomenon combined for the Next-5 largest economies. This study also considers additional macroeconomic indicators like FDI inflow or the role of the urban population in context. Consequently, the present study opens room for future studies. A complimentary validation of various empirical evidence can be carried out to offer a broader view of environmental problems, energy efficiency or sustainable economic growth for other blocs and divides.
